# Peroxiredoxin II regulates exosome secretion from dermal mesenchymal stem cells through the ISGylation signaling pathway

**DOI:** 10.1186/s12964-023-01331-w

**Published:** 2023-10-20

**Authors:** Ying-Hao Han, Ying-Ying Mao, Kyung Ho Lee, Hee Jun Cho, Nan-Nan Yu, Xiao-Ya Xing, Ai-Guo Wang, Mei-Hua Jin, Kwan Soo Hong, Hu-Nan Sun, Taeho Kwon

**Affiliations:** 1 College of Life Science and Technology, Heilongjiang Bayi Agricultural University, Daqing, Heilongjiang 163319 P.R. China; 2 KRIBB School of Bioscience, University of Science and Technology, Daejeon, 34113 Republic of Korea; 3 Chemical Biology Research Center, Korea Research Institute of Bioscience and Biotechnology (KRIBB), Cheongju, Chungbuk, 28116 Republic of Korea; 4 Immunotherapy Research Center, Korea Research Institute of Bioscience and Biotechnology (KRIBB), Daejeon, 34141 Republic of Korea; 5 Laboratory Animal Center, Dalian Medical University, Dalian, 116041 P.R. China; 6 Research Center for Bioconvergence Analysis, Korea Basic Science Institute, Cheongju, Chungbuk 28119 Korea; 7 Primate Resources Center, Korea Research Institute of Bioscience and Biotechnology (KRIBB), Jeongeup, Jeonbuk 56216 Republic of Korea

**Keywords:** Peroxiredoxin II, Dermal mesenchymal stem cells, Exosome, ISGylation

## Abstract

**Background:**

Exosomes are small extracellular vesicles that play important roles in intercellular communication and have potential therapeutic applications in regenerative medicine. Dermal mesenchymal stem cells (DMSCs) are a promising source of exosomes due to their regenerative and immunomodulatory properties. However, the molecular mechanisms regulating exosome secretion from DMSCs are not fully understood.

**Results:**

In this study, the role of peroxiredoxin II (Prx II) in regulating exosome secretion from DMSCs and the underlying molecular mechanisms were investigated. It was discovered that depletion of Prx II led to a significant reduction in exosome secretion from DMSCs and an increase in the number of intracellular multivesicular bodies (MVBs), which serve as precursors of exosomes. Mechanistically, Prx II regulates the ISGylation switch that controls MVB degradation and impairs exosome secretion. Specifically, Prx II depletion decreased JNK activity, reduced the expression of the transcription inhibitor Foxo1, and promoted miR-221 expression. Increased miR-221 expression inhibited the STAT signaling pathway, thus downregulating the expression of ISGylation-related genes involved in MVB degradation. Together, these results identify Prx II as a critical regulator of exosome secretion from DMSCs through the ISGylation signaling pathway.

**Conclusions:**

Our findings provide important insights into the molecular mechanisms regulating exosome secretion from DMSCs and highlight the critical role of Prx II in controlling the ISGylation switch that regulates DMSC-exosome secretion. This study has significant implications for developing new therapeutic strategies in regenerative medicine.

Video Abstract

**Supplementary Information:**

The online version contains supplementary material available at 10.1186/s12964-023-01331-w.

## Introduction

Stem cell-derived exosome therapy has been developed as a potential cell-free therapeutic strategy for modulating inflammation and repairing tissue damage. Exosomes derived from mesenchymal stem cells (MSCs) have strong regenerative, reparative, and protective capabilities against various diseases [1], making them important targets for disease mechanism research and effective candidates for cell-free therapy. Exosomes are nano-sized particles with diameters ranging from 40 to 200 nanometers. They play a critical role in intercellular communication and the transport of various molecules, including proteins, microRNAs (miRNAs), and messenger RNAs (mRNAs). These vesicles emerge from late endosomes, which transform into multivesicular bodies (MVBs) through multiple membrane invaginations. These invaginations enclose selected molecular cargo, leading to the formation of intraluminal vesicles (ILVs) [2]. Some MVBs fuse with the plasma membrane under the regulation of the RAB and SNARE families, releasing ILVs as exosomes into the extracellular environment [3, 4]. However, not all generated MVBs are secreted to form exosomes. TSG101 protein on some MVBs binds to ISG15, causing MVB aggregation and subsequent lysosomal degradation, thereby reducing exosome secretion [5]. The molecular mechanisms directly regulating exosome secretion and trafficking have been extensively studied, but the upstream regulators of exosome formation and secretion are not well understood.

Interferon-stimulated gene 15 (ISG15), the initial Ubiquitin-like (Ubl) protein, forms covalent bonds with target proteins via UBA7, UBE2L6, and HERC6 enzymes. This process, termed ISGylation, is triggered by type I interferons [6]. The dimerization of IFNAR1 and IFNAR2 activates the JAK-STAT pathway, resulting in the creation of ISGF3 complex and subsequent transcriptional activation of ISG15 and its associated enzymes. Beyond its antiviral roles, ISGylation also influences DNA repair, autophagy, protein translation, and exosome secretion [7]. ISGylation is a post-translational ubiquitin-like modification that is considered to be one of the signals regulating multivesicular body (MVB) degradation. It has a crucial impact on regulating exosome secretion by controlling the interferon (IFN) signaling pathway [5]. Stem cells express a subset of genes previously classified as IFN-stimulated genes (ISGs), but their expression is intrinsic because stem cells are refractory to IFN [8]. Therefore, clarifying the regulatory mechanism of ISG expression in stem cells and its role in regulating exosome secretion is critical for stem cell exosome therapy. Our previous study showed that the impact of exosomes derived from dermal mesenchymal stem cells (DMSCs) on skin wounds was enhanced after Prx II knockout [9], suggesting that Prx II may be a key regulator of DMSC-exosome (DMSC-Exos) secretion. All of the above evidence suggests that Prx II has a potential role in regulating exosome secretion.

To demonstrate that Prx II is a key regulator of DMSC-exosome (DMSC-Exos) secretion, we aimed to elucidate the molecular mechanisms by which Prx II regulates the ISGylation switch that controls MVB degradation and impairs exosome secretion. The present study aimed to elucidate the role of peroxiredoxin II (Prx II) in regulating exosome secretion from DMSCs. Our findings indicate that depletion of Prx II leads to a significant reduction in exosome secretion from DMSCs and an increase in the number of intracellular multivesicular bodies (MVBs), which are precursors of exosomes. Mechanistically, Prx II regulates the ISGylation switch that controls MVB degradation and impairs exosome secretion. Specifically, Prx II depletion decreased JNK activity, reduced the expression of the transcription inhibitor Foxo1, and promoted miR-221 expression. Increased miR-221 expression inhibited the STAT signaling pathway, thus downregulating the expression of ISGylation-related genes involved in MVB degradation.

In summary, this study aimed to investigate the regulatory role of Prx II in the ISGylation and MSC-derived exosomes in DMSCs and elucidate its potential molecular mechanisms. We highlighted the critical role of Prx II in controlling the ISGylation switch that regulates DMSC-Exo secretion. These findings have important implications for developing new therapeutic strategies for the treatment diseases.

## Materials and methods

### Cell culture

Primary dermal mesenchymal stem cells (DMSCs) were obtained from the dermis of wild-type and Prx II knockout 129/SvJ mice. All mice for this study were purchased from Dalian Medical University. Mice were kept under the temperature at 20–22℃, the humidity 50–60% and the 12-h-dark/ light cycles conditions. DMSCs were isolated and identified as previously described [10]. The cells were cultured in Dulbecco’s modified Eagle’s medium (GibcoBRL, Grand Island, NY, USA) and Nutrient Mixture F-12 (GibcoBRL, Grand Island, NY, USA) containing 10% fetal bovine serum (Solarbio Life Sciences, Beijing, China) and 100 U/mL penicillin-streptomycin (Solarbio Life Sciences) at 37 °C in a 5% CO_2_ incubator. The culture medium was replaced every day, and the cells were subcultured *via* treatment with 0.25% trypsin–Ethylene Diamine Tetraacetic Acid (EDTA; Solarbio Life Sciences) at 90% confluency.

### Isolation and characterization of DMSC-exosomes

DMSCs were cultured at a cell density of 2 × 10^6^ cells and mixed in DMSCs culture medium. The cell mixture was then inoculated into 10 cm Petri dishes. The prepared DMSCs medium was centrifuged at 120,000 ×g overnight to remove exosomes from the medium. When the cell density reached 80%, the DMSCs culture medium devoid of exosome was replaced. After 24 h of incubation, the culture medium was collected.

Exosomes were separated from the collected culture medium using sequential centrifugation at 4℃. The following centrifugation steps were performed: first, centrifugation at 1500 ×g for 15 min at 4℃; then, the supernatant was collected and centrifuged at 10,000 ×g for 30 min at 4℃; subsequently, the supernatant was collected and centrifuged at 14,000 ×g for 30 min at 4 °C. The resulting supernatant was further centrifuged at 120,000 ×g for 120 min at 4 °C. Discard the supernatant and resuspend the exosome pellet in 1×PBS buffer. This resuspended pellet was then subjected to another round of centrifugation at 120,000 ×g for 60 min at 4℃. The supernatant was carefully discarded, and the exosome pellet was resuspended in 100 µl 1×PBS buffer, transferred to a 1.5 ml centrifuge tube, and stored at -80℃.

To assess the ultrastructure and size distribution of the exosomes, nanoparticle tracking analysis (NTA) was conducted using laser scattering microscopy. Particle Metrix (Germany) and ZetaView 8.04.02 SP2 software were used for this analysis (XP Biomed, Shanghai, China). For the identification of vesicular markers, exosomes were lysed using RIPA lysate and performed by western blotting (Yanzai Biotechnology, Shanghai, China).

### Determination of total protein concentration of exosomes from DMSC

According to the manufacturer’s instructions of the BCA Protein Assay Kit (Solarbio, China, PC0020), equal volumes of Prx II^−/−^ DMSCs-Exos and Prx II^+/+^ DMSCs-Exos were mixed with the BSA work reagent and incubated at 37℃ for 15–30 min. The OD values of the samples were measured using a Microplate reader (Tecan Infinite 200 Pro) at a wavelength of 562 nm and by using the standard curve to calculate the protein concentration of exosome.

### RNA extraction and qRT-PCR

RNA was extracted using TRIzol® reagent (Sigma-Aldrich, St. Louis, MO, USA) and sequenced using HiSeq (Genminix, Shanghai, China). Total RNA was extracted from the lungs or DMSCs using TRIzol^®^ reagent (Invitrogen, Carlsbad, CA, USA) according to the manufacturer’s instructions and reverse transcribed to cDNA using the inNova Uscript II All in One First Strand cDNA Synthesis SuperMix kit (Innovagene Biotech, Hunan, China). qRT-PCR was performed on a CFX96 real-time PCR system (Bio-Rad, CA, USA) using the inNova Taq SYBR Green qPCR Mix (Innovagene Biotech,), with β-actin as an endogenous control. Primers were designed and synthesized by Sangon Biotech (Shanghai, China) and are listed in Table 1. miRNA was reverse transcribed to cDNA using the miRNA First Strand cDNA Synthesis (Tailing Reaction) kit (Sangon Biotech) according to the manufacturer’s instructions. qRT-PCR was performed on a CFX96 real-time PCR system (Bio-Rad) using the MicroRNA qPCR Kit (SYBR Green Method) (Sangon Biotech), with U6 as an endogenous control. Primers were designed and synthesized by Sangon Biotech and are listed in Table 2. Data are presented as relative Ct values, and relative expression levels were calculated using the 2- ^ΔΔCt^ method.

**Table 1:** mRNA primer sequences

	Forward primer(5’-3’)	Reversed primer(3’-5’)
**ISG15**	**TGCCTGCAGTTCTGTACCAC**	**AGTGCTCCAGGACGGTCTTA**
**Rab27b**	**AAGGCAGACCTACCAGATCAGAG**	**TTCTCCACACACTGTTCCATTCG**
**UBA7**	**GTTAATCATGCTCGGATCAAGC**	**AGATAGCTGTGACGAAAGGTAC**
**UBE2L6**	**CACTTTGAGATTCACCACCAAG**	**TGTAAGGCTTCCAGTTCTCATT**
**HERC6**	**GAGGAGAGACCAGAGTACCGAAAGG**	**CAGACAGCCACAGAGTGTTCCTTC**
**STAT1**	**TCACAGTGGTTCGAGCTTCAG**	**CGAGACATCATAGGCAGCGTG**
**STAT2**	**GTTACACCAGGTCTACTCACAGA**	**TGGTCTTCAATCCAGGTAGCC**
**IRF9**	**CGCTGCTGCTCACCTTCATCTATG**	**TCCATGCTGCTCTCCGAGTCTG**
**β-actin**	**GACGGCCAGGTCATCACTATTG**	**AGGAAGGCTGGAAAAGAGCC**

**Table 2:** miRNA primer sequences

	Forward primer(5’-3’)		Forward primer(5’-3’)
**miR-27a-3p**	**TTCACAGTGGCTAAGTTCCGC**	**miR-152-3p**	**TCAGTGCATGACAGAACTTGG**
**miR-27b-3p**	**TTCACAGTGGCTAAGTTCTGC**	**miR-212-3p**	**TAACAGTCTCCAGTCACGGCCA**
**miR-19a-3p**	**TGTGCAAATCTATGCAAAACTGA**	**miR-214-3p**	**ACAGCAGGCACAGACAGGCAGT**
**miR-19b-3p**	**TGTGCAAATCCATGCAAAACTGA**	**miR-291a-3p**	**AAAGTGCTTCCACTTTGTGTGC**
**miR-15a-5p**	**TAGCAGCACATAATGGTTTGTG**	**miR-292a-3p**	**AAAGTGCCGCCAGGTTTTGAGTGT**
**miR-15b-5p**	**TAGCAGCACATCATGGTTTACA**	**miR-294-3p**	**AAAGTGCTTCCCTTTTGTGTGT**
**miR-16-5p**	**TAGCAGCACGTAAATATTGGCG**	**miR-295-3p**	**AAAGTGCTACTACTTTTGAGTCT**
**miR-155-5p**	**TTAATGCTAATTGTGATAGGGGT**	**miR-302a-3p**	**TAAGTGCTTCCATGTTTTGGTGA**
**miR-103-3p**	**AGCAGCATTGTACAGGGCTATGA**	**miR-1983**	**CTCACCTGGAGCATGTTTTCT**
**miR-132-3p**	**TAACAGTCTACAGCCATGGTCG**	**miR-412-3p**	**TTCACCTGGTCCACTAGCCG**
**miR-148a-3p**	**TCAGTGCACTACAGAACTTTGT**	**miR-665-3p**	**ACCAGGAGGCTGAGGTCCCT**
**miR-148b-3p**	**TCAGTGCATCACAGAACTTTGT**		

### mRNA sequencing

Prx II^+/+^ and Prx II^−/−^ DMSCs were seeded in a 10 cm culture dish. When the cell confluence reached 80%, 1 ml of Trizol was added to lyse the cells. Prx II^+/+^ and Prx II^−/−^ DMSCs RNA was extracted from Prx II^+/+^ and Prx II^−/−^ DMSCs and reverse transcribed into cDNA. Differential gene analysis was performed using the Illumina sequencer (Gminix, Shanghai, China).

### Electron microscopy and MVB quantification

DMSCs cultured on dishes were washed with PBS and fixed with 2.5% glutaraldehyde overnight at 4 °C. The cells were post-fixed with 1% osmium tetroxide in phosphate buffer for 2 h, dehydrated using a graded ethanol series, and embedded in Epon812. Ultrathin Sect. (70 nm) were prepared, stained with uranyl acetate and lead citrate, and examined using transmission electron microscopy (JEOL, JEM1400, Japan) (Beijing Zhongke Baice Technology Service, Beijing, China).

MVBs were identified and counted based on their morphology, with only discrete ILVs. At least 20 MVBs per experiment were analyzed using separate cells. Box scatter plots were constructed using GraphPad Prism software (GraphPad Software Inc., La Jolla, CA, USA), and statistical analyses were performed using SPSS software (SPSS Inc., Chicago, IL, USA). Data are presented as the mean ± standard deviation, and statistical significance was set at *p* < 0.0001.

### Lentivirus-mediated re-expression of Prx II

To construct lentiviral vectors expressing GFR-tagged Prx II, murine full-length Prx II cDNA was used as a template to clone Prx II into the LV5. Lentiviruses were purchased from Suzhou GenePharma (Shanghai, China). DMSCs were grown in six-well plates to 80% confluency, after which LV-Prx II was transduced at a multiplicity of infection of 60 using polybrene (10 g/mL), and an empty vector was used as a control (GenePharma). After 5–7 d of puromycin selection at a final concentration of 2 µg/mL, Prx II-reexpressing (wild-type [WT]) and control (NC) cells were harvested. Prx II re-expression was detected via western blotting, flow cytometry, and fluorescence imaging.

### Western blotting

The protein concentration was determined using the Thomas Brilliant Blue method, employing a standard of 20 µg total protein. The procedure involved stepwise addition of 5× upsampling buffer, the appropriate volume of protein samples, and sterile deionized mix. Total proteins from DMSC cell lysates were subjected to sodium dodecyl sulfate-polyacrylamide gel electrophoresis using a 12–15% gel and transferred to nitrocellulose membranes (Millipore, Bedford, MA, USA). The membranes were washed five times with Tris-buffered saline (TBS). The following primary antibodies were used: anti-CD63 (Sangon Biotech, D260973), anti-EEA1 (Sangon Biotech, D163757), anti-Prx II (AbFrontier, Seoul, Republic of Korea, LF-MA0114), anti-ISG15 (Sangon Biotech, D225264), anti-UBA7 (Sangon Biotech, D223465), anti-UBE2L6 (Sino Biological, Beijing, China, 12,641-RP02), anti-HERC6 (Bioss, Beijing, China, bs-15463R), anti-Rab27b (Bioss, bsm-51,331 M), anti-ISG15 (Solarbio Life Sciences, K002371P), anti-TSG101 (Bioss, bs-1365R), anti-STAT1 (Sangon Biotech, D220084), anti-P-STAT1 (Santa Cruz Biotechnology, Santa Cruz, CA, USA, sc-8394), anti-STAT2 (Sangon Biotech, D261445), anti-P-STAT2 (Bioss, bs-3428R), anti-STAT2 (AtaGenix, Wuhan, China, ATA38071), and anti-IRF9 (Sangon Biotech, D220878). The following secondary antibodies were used: goat anti-mouse (Sangon Biotech, Shanghai, China, D110087), goat anti-rabbit (Sangon Biotech, D111018), and donkey anti-goat (Sangon Biotech, D110120). Normalization was performed with β-actin or α-tubulin (both from Santa Cruz Biotechnology, sc-47,778) as an internal reference. Protein bands were visualized using Alpha View Software (AlphaView, USA, sc-8035) and analyzed using ImageJ software (National Institute of Health, Bethesda, MD, USA).

### Cell fractionation

Cells were lysed in Triton buffer containing 10 mM Tris-HCl, pH 8, 100 mM NaCl, 1 mM EDTA, 0.5% NP40, and a cocktail of protease inhibitors for 30 min at 4 °C. The lysates were centrifuged at 13,200 g for 20 min. The supernatants were used as detergent-soluble fractions. The pellets were used as detergent-insoluble fractions and suspended in a buffer containing 7 M urea, 2 M thiourea, 4% CHAPS, and 40 mM Tris. Equal amounts of protein from the soluble and insoluble fractions were used for immunoblotting as described above.

### Bioinformatics analysis

The protein-protein interaction network was analyzed using the STRING database [11]. Functional interactions between proteins were analyzed to elucidate the mechanisms of ISGylation. the target miRNAs of STAT1 and STAT2 were predicted using the online databases miRDB [12], StarBase [13], and TarBase [14], and a weien picture was drawn using bioinformatics to search for common predictive target miRNAs. Using the JASPAR database (default threshold score of 85.0) [15] we identified four potential binding sites for the *miR-221* promoter in the coding region of *Foxo1*.

### Transfection of miR-221 inhibitor and SP600125 treatment

Prx II^+/+^ and Prx II^−/−^ DMSCs were cultured in six-well plates at a density of 2 × 10^5^ cells/well. At 40% confluency, the cells were transfected with the miR-221 inhibitor (200 nM) or negative control (RiboBio, Guangzhou, China) for 48 h using a RiboFect CP Transfection Kit (RiboBio) according to the manufacturer’s instructions. The cell lysates were used for western blot analysis.

Prx II^+/+^ and Prx II^−/−^ DMSCs were cultured in 6-well plates at a density of 2 × 10^5^ cells/well and incubated for 24 h. They were treated with the JNK inhibitor SP600125 (25 μm) for 48 h. Nuclear fractions were isolated using a nuclear extraction kit (Solarbio Life Sciences) for nuclear isolation via western blotting as described previously, and miR-221 was detected via qRT-PCR.

### JNK activity assay

*Prx II* ^*+/+*^ and *Prx II* ^*−/−*^ DMSCs were cultured in 100 mm culture dishes (NEST Biotechnology, Wuxi, Jiangsu, China). JNK activity was assayed using a JNK kinase activity assay kit (GenMed, China), according to the manufacturer’s instructions. Absorbance was measured at 340 nm using a microplate reader (AOE Instruments, Shanghai, China).

### Statistical analysis

All data are presented as mean ± standard error (SE) from at least three independent experiments and analyzed by the GraphPad Prism 8.0 software (GraphPad Software Inc., La Jolla, CA, USA). Comparison between two groups was performed by Student’s t test. A *P*-value of < 0.05 was considered to reflect a statistically significant difference.

## Results

### Prx II depletion promotes exosome secretion from DMSCs

**Fig. 1:**
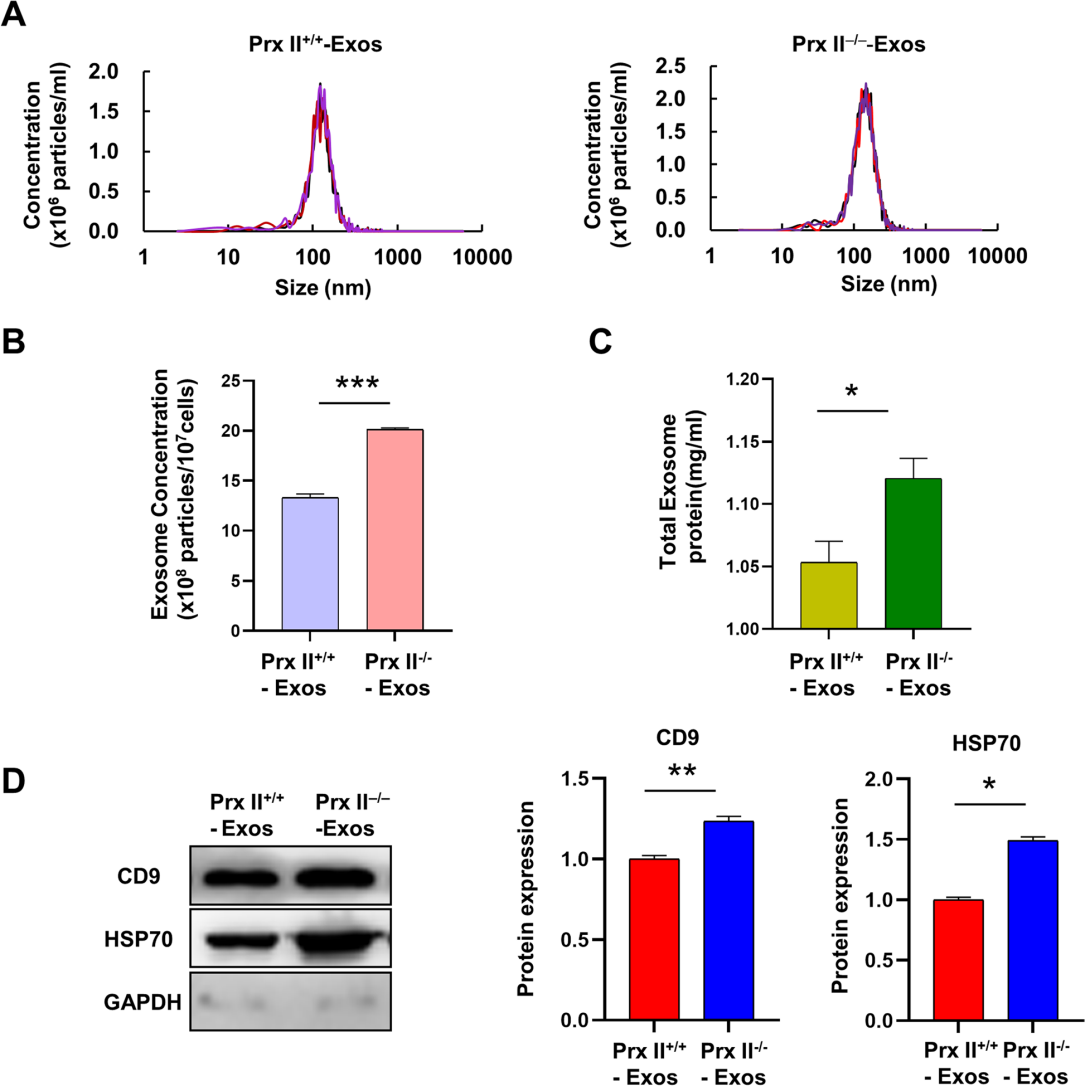
Prx II regulates exosome secretion in DMSCs. **A** Exosome size was analyzed by nanoparticle tracking analysis (NTA). Median statistics revealed that the diameter of *Prx II* ^*+/+*^ DMSCs-Exos was 142.5 nm, while the diameter of *Prx II* ^*−/−*^ DMSCs- Exos was 152.5 nm. **B** NTA of the number of exosomes released by two cell types with equal cell volume. **C** The concentration of secreted protein was determined by bicinchoninic acid assay. **D** Western blot analysis of exosomes purified *via* serial ultracentrifugation of culture supernatants of equal numbers of *Prx II* ^*+/+*^ and *Prx II* ^*−/−*^ DMSCs using the vesicular markers CD9 and HSP70. Quantification of protein contents in Prx II^+/+^ and Prx II^−/−^Exos. *P < 0.05, **P < 0.01, ***P < 0.001 as determined by two-tailed t test

To investigate the role of Prx II in DMSC exosome production, exosomes were isolated from both *Prx II* ^*+/+*^ DMSCs and Prx II knockout DMSCs (*Prx II* ^*−/−*^ DMSCs). Exosomes are characterized by their cup-shaped morphology and range in size from 40 to 200 nm [5]. Using nanoparticle tracking analysis (NTA), we observed that the size distribution of exosomes remained unchanged following the deletion of Prx II. The diameters of *Prx II* ^*−/−*^ DMSCs-Exos and the WT group (*Prx II* ^*+/+*^ DMSCs-Exos) were found to be similar (Fig. 1A). In our previous study [9], NTA and electron microscopic analyses unveiled that the majority of vesicles pelleted through ultracentrifugation exhibited sizes consistent with those typically associated with exosomes (ranging from 40 to 200 nm). Hence, we employ the term “exosomes” in this paper, although the isolates may contain other Extracellular Vesicles (EVs) sub-populations as well. However, a significant increase in the number of exosome particles secreted by *Prx II* ^*−/−*^ DMSCs compared to control cells was observed, as confirmed by NTA analysis of DMSC-Exos (Fig. 1B) and measurement of protein concentrations (Fig. 1C). Furthermore, examination of exosome protein markers revealed that Prx II depletion led to an increase in the levels of CD9 and HSP70 in *Prx II* ^*−/−*^ Exos (Fig. 1D). These results suggest that Prx II plays a critical role in regulating exosome secretion from DMSCs, independent of exosome size.

### Prx II depletion increases the number of MVBs in DMSCs

**Fig. 2:**
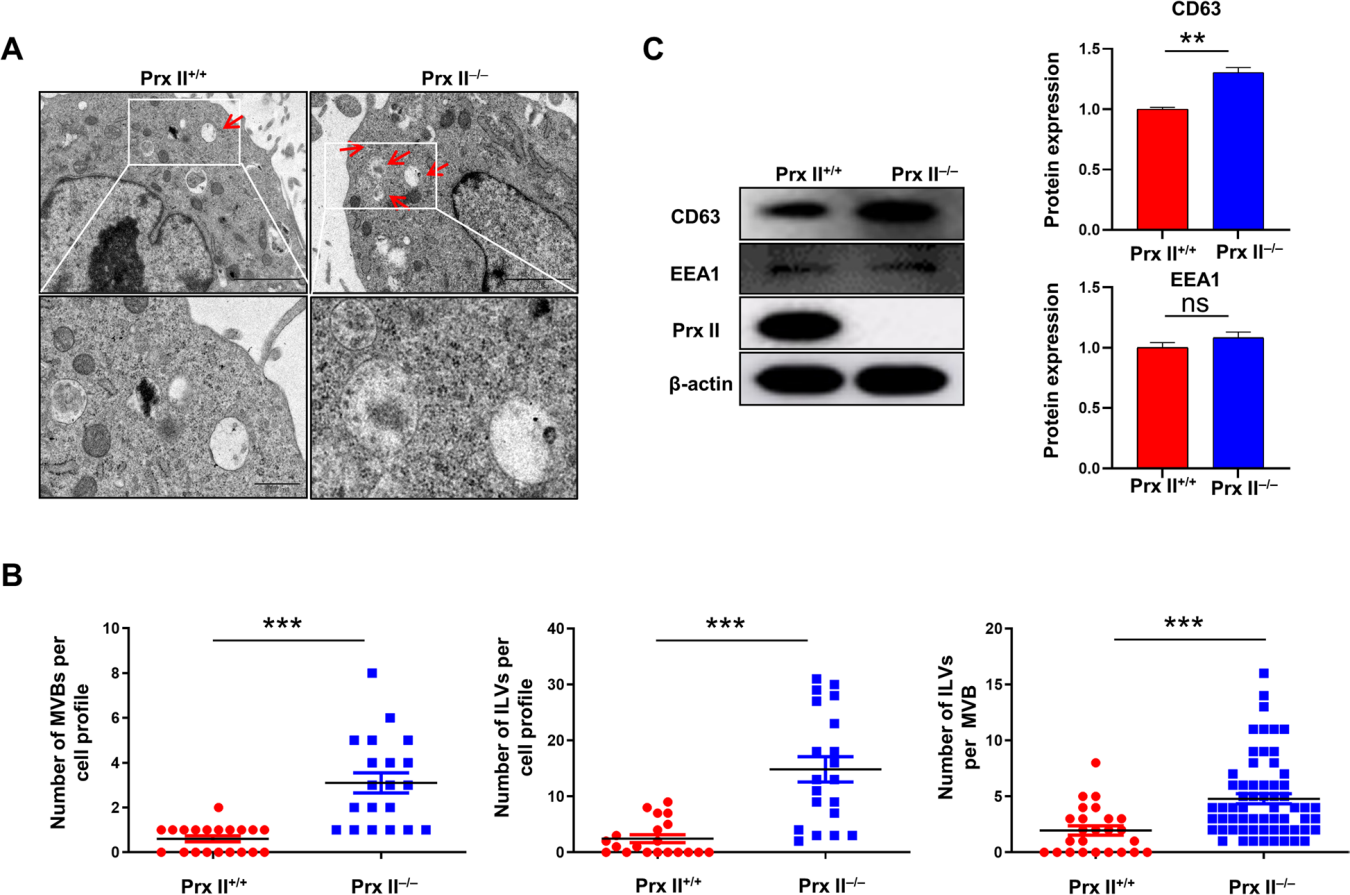
Prx II depletion increases MVB number in DMSCs. **A** Electron micrographs showing MVBs in *Prx II* ^*+/+*^ and *Prx II* ^*−/−*^ DMSCs. Red arrows indicate MVBs containing typical intraluminal vesicles (ILVs). **B** Determination of the numbers of MVBs, ILVs, and ILV per MVB in 20 fields per condition. **C** Western blot analysis of CD63 and EEA1 in Prx *II* ^*−/−*^ DMSCs and control cells. Quantification of CD63 and EEA1 expression. **P < 0.01, ***P < 0.001 as determined by two-tailed t test

Exosomes are small extracellular vesicles that originate from MVBs, which are intracellular organelles containing multiple ILVs. MVBs release their ILVs as exosomes when they fuse with the plasma membrane. MVBs primarily characterized by the presence of numerous smaller vesicles enclosed within their structure. These MVBs were observed to exhibit a collective size ranging from 100 to 600 nm, accommodating vesicles with individual diameters of up to 50 nm [16]. To investigate the role of Prx II in this process, we studied MVBs and ILVs in DMSCs. Electron microscopic analysis revealed a significant increase in the number of MVBs per cell after Prx II knockout, and interestingly, the number of ILVs per cell also increased (Supplementary Fig. 1 and Fig. 2A and B). The expression of CD63, a common MVB membrane protein, was examined in *Prx II* ^*−/−*^ and *Prx II* ^*+/+*^ DMSCs and found to be higher in MVBs of *Prx II* ^*−/−*^ DMSCs than in those of *Prx II* ^*+/+*^ DMSCs (Fig. 2C). Since MVBs are formed by inward budding of the early endosomal membrane, the increased number of MVBs could be due to either a massive increase in MVB formation or inhibition of MVB degradation. However, we observed no significant alterations in the expression of the early endosome marker EEA1 in *Prx II* ^*−/−*^ and *Prx II* ^*+/+*^ DMSCs, suggesting that Prx II may affect MVB degradation rather than MVB formation (Fig. 2C). Taken together, these results suggest that Prx II depletion promotes DMSC-Exo secretion by increasing MVBs.

### Prx II depletion promotes exosome secretion by regulating ISGylation

**Fig. 3:**
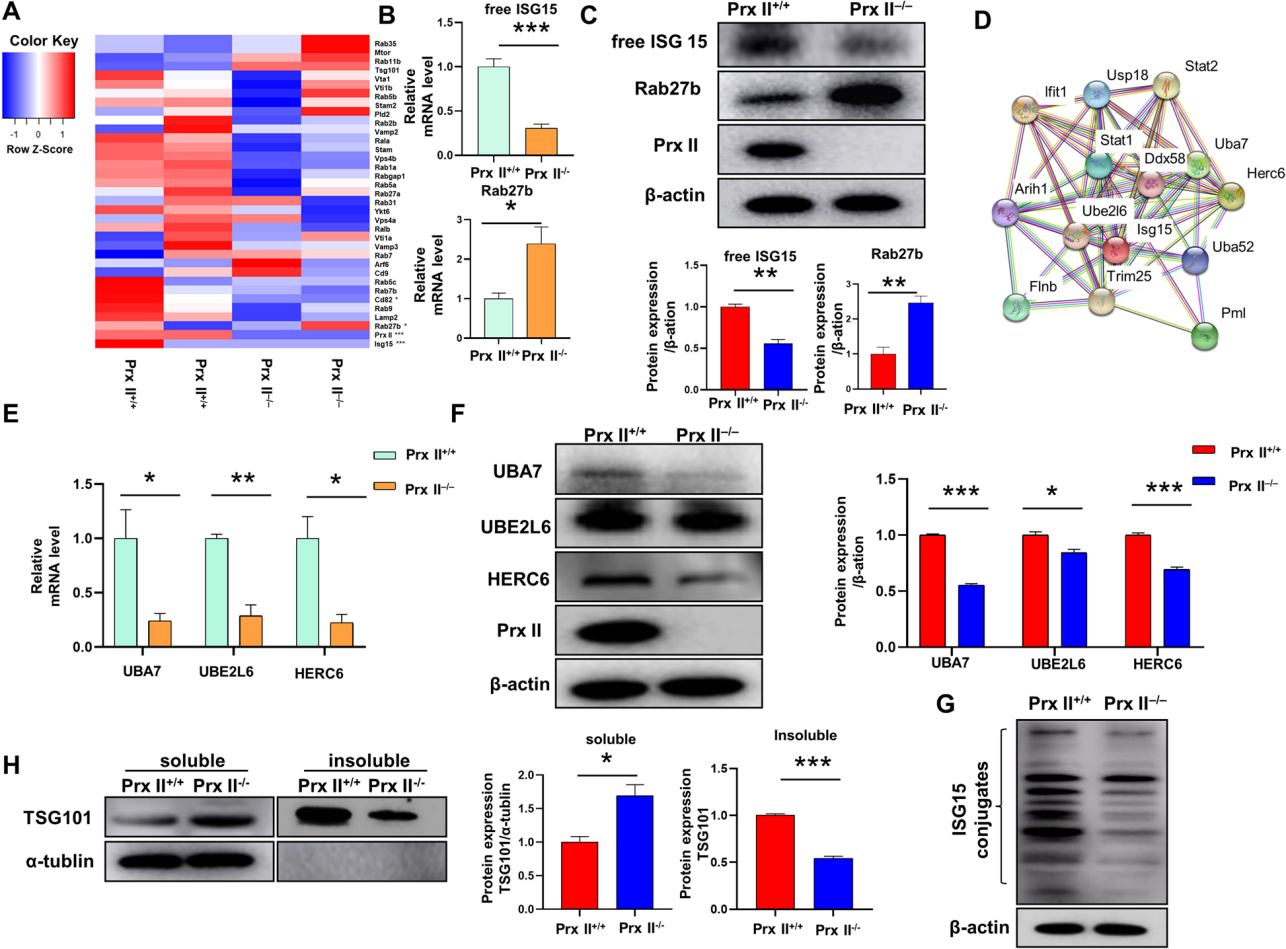
Prx II depletion promotes exosome secretion by regulating ISGylation. **A** Heat map of proteins involved in exosome biogenesis or secretion identified *via* RNA-sequencing of *Prx II* ^*+/+*^ and *Prx II* ^*−/−*^ DMSCs. Red and blue hues represent upregulated and downregulated mRNA, respectively. **B** mRNA expression of ISG15 and Rab27b in *Prx II* ^*+/+*^ and *Prx II* ^*−/−*^ DMSCs was measured by qRT-PCR. **C** Protein expression of ISG15 and Rab27b was detected by western blotting of *Prx II* ^*+/+*^ and *Prx II* ^*−/−*^ DMSCs. Quantification of ISG15 and Rab27b expression. **D** Proteins known to interact with ISGylation-related genes with high confidence are shown. The image was created using the STRING proteomics database. **E** qRT-PCR analysis of UBA7, UBE2L6, and HERC6 levels in *Prx II* ^*−/−*^ DMSCs and control cells, with β-actin as the control. **F** UBA7, UBE2L6 and HERC6 protein expression levels and quantitative analysis results **G** The expression of TSG101 in soluble and insoluble components level detection and quantitative. *P < 0.05, **P < 0.01, ***P < 0.001 as determined by two-tailed t test

In this study, we aimed to investigate the molecular mechanisms underlying the regulation of exosome secretion by Prx II in DMSCs. RNA-sequencing was conducted to analyze proteins involved in exosome formation or secretion, and significant alterations in the mRNA levels of Rab27b and ISG15 were observed in Prx II knockout DMSCs (Fig. 3A). This result was further supported by qRT-PCR and western blot analysis (Fig. 3B and C). Notably, ISG15 undergoes enzymatic conjugation with TSG101 on the MVB membrane, a process known as ISGylation, under the continuous action of E1 activated enzyme (UBA7), E2 ubiquitin-conjugating enzyme (UBE2L6), and E3 ligase (HERC6). A search for interactive partners of ISG15 in the publicly available STRING database showed high confidence in the interaction between ISG15, UBA7, UBE2L6, and HERC6 in the ISGylation pathway (Fig. 3D). Downregulation of all ISGylated components at the mRNA level, including ISG15, UBA7, UBE2L6, and HERC6, was observed in Prx II knockout DMSCs, which was further supported by western blot analysis (Fig. 3E and F). In *Prx II* ^*−/−*^ DMSCs, we observed a considerable decrease in both free and conjugated forms of ISG15 (Fig. 3C and G). ISGylation induces the aggregation and degradation of TSG101, which participates in the regulation of lysosomal MVB degradation [5]. Interestingly, ISGylation induced a decrease in TSG101 accumulation in the insoluble fractions of *Prx II* ^*−/−*^ DMSCs (Fig. 3H). These results suggest that Prx II knockout can inhibit the expression of ISGylation-related genes, lessen TSG101 aggregation on the MVB membrane, and reduce MVB degradation, which indirectly leads to an increase in Rab27b secreted by transferred MVB to the outside of the cells and promotes exosome secretion. Overall, these findings provide new insights into the regulatory mechanisms of exosome secretion in DMSCs and highlight the critical role of Prx II in this process.

### Prx II depletion downregulates ISGylation by regulating the STAT signaling pathway

**Fig. 4:**
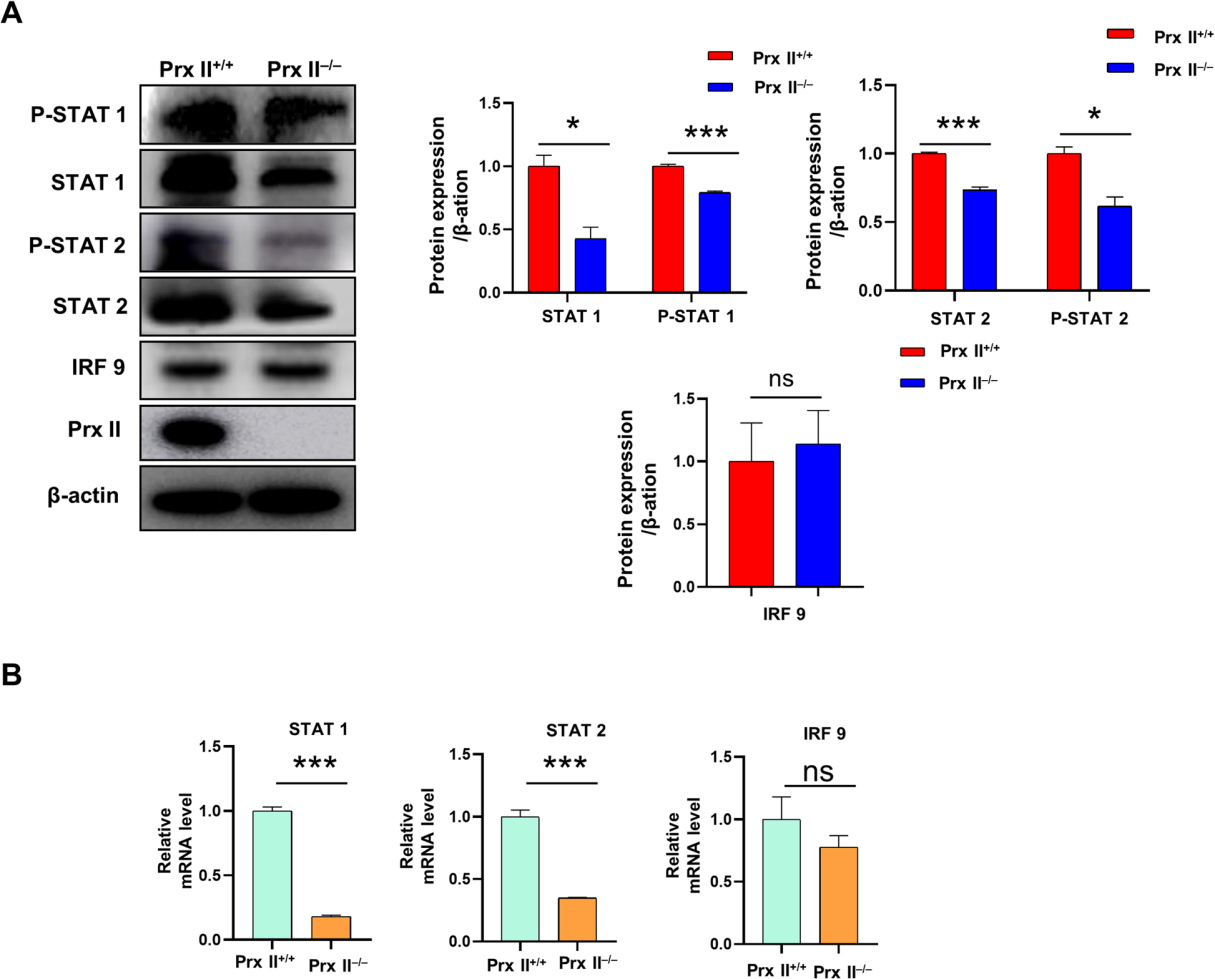
Prx II depletion downregulates ISGylation by regulating the STAT signaling pathway. **A** Western blot analysis of phospho-STAT1, STAT1, phospho-STAT2, STAT2, and IRF9 in *Prx II* ^*+/+*^ and *Prx II* ^*−/−*^ DMSCs. Quantification of phospho-STAT2, STAT2, and IRF9 expression. **B** mRNA levels of STAT1, STAT2, and IRF9 in *Prx II* ^*+/+*^ and *Prx II* ^*−/−*^ DMSCs were determined using qRT-PCR. *P < 0.05, ***P < 0.001 as determined by two-tailed t test

The study revealed that upon IFN stimulation, phosphorylated STAT1 and STAT2 form the ISGF3 complex with IRF9, translocating to the nucleus and inducing the expression of ISGylation-related genes, including ISG15, UBA7, UBE2L6, and HERC6 [17]. To investigate the molecular mechanism of Prx II in regulating ISGylation, we initially examined the protein expression levels of the upstream molecules of ISGylation, namely STAT1, P-STAT1, STAT2, P-STAT2, and IRF9. The results indicated that the levels of P-STAT1 and P-STAT2 were significantly reduced in *Prx II* ^*−/−*^ DMSCs (Fig. 4A). Interestingly, we also observed decreased expression levels of STAT1 and STAT2 in *Prx II* ^*−/−*^ DMSCs, while IRF9 levels did not exhibit a significant difference (Fig. 4A). Conversely, the reintroduction of Prx II into *Prx II* ^*−/−*^ DMSCs (WT group) led to an increase in the expression levels of STAT1, P-STAT1, STAT2, and P-STAT2 (Supplementary Figs. 2 and 3A). This implies that the decrease in P-STAT1 and P-STAT2 protein levels caused by Prx II is mediated by the decrease in STAT1 and STAT2 protein levels.

Furthermore, we examined the transcription levels of STAT1 and STAT2 and found that they were also significantly downregulated in *Prx II* ^*−/−*^ DMSCs (Fig. 4B), whereas they were partially restored in WT cells (Supplementary Fig. 3B). It is well established that the induction of ISGylation-related gene expression in response to IFN action is achieved through phosphorylated STAT1 and STAT2, independent of total protein expression levels. This leads us to hypothesize that Prx II activates the STAT signaling pathway by inhibiting the transcription levels of STAT1 and STAT2, thereby downregulating the expression of ISGylation-related genes.

### Depletion of prx II inhibits STAT signaling pathway by regulating Foxo1-induced expression of miR-221

**Fig. 5:**
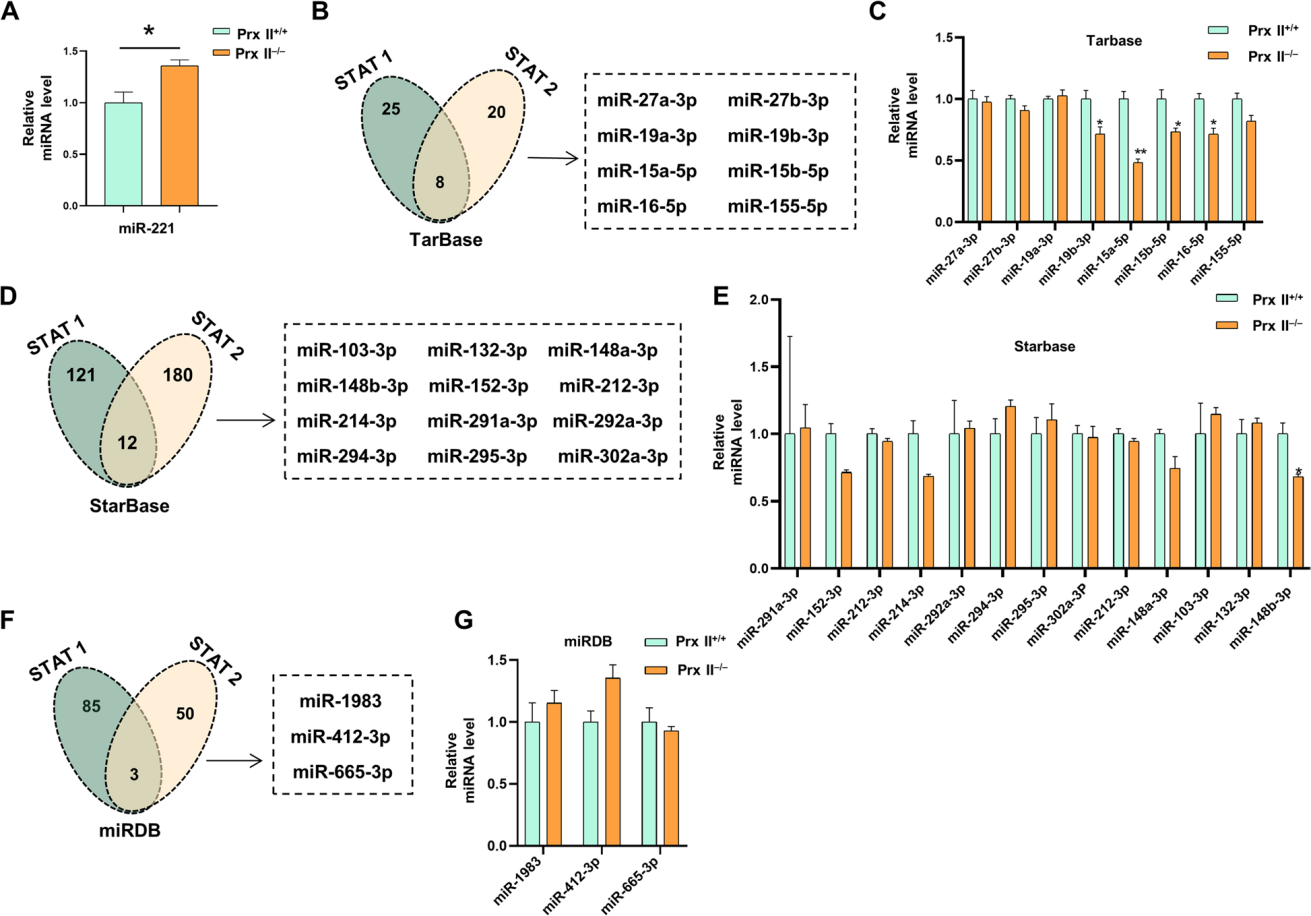
Depletion of Prx II inhibits STAT signaling pathway by regulating Foxo1-induced expression of miR-221. **A** Quantification of miR-221 expression in *Prx II* ^*+/+*^ and *Prx II* ^*−/−*^ DMSCs using qRT-PCR analysis. **B** Venn diagrams of the target miRNAs of STAT1 and STAT2 predicted by TarBase. **C** qRT-PCR was used to measure the expression of the various miRNAs of TarBase in *Prx II* ^*+/+*^ and *Prx II* ^*−/−*^ DMSCs. **D** Venn diagrams of the target miRNAs of STAT1 and STAT2 predicted by StarBase. **E** qRT-PCR was used to measure the expression of the various miRNAs of starBase. **F** Venn diagrams of the target miRNAs of STAT1 and STAT2 predicted by miRDB. **G** qRT-PCR was used to measure the expression of the various miRNAs of miRDB. *P < 0.05, **P < 0.01, as determined by two-tailed *t* test

Previous studies have demonstrated that Prx II deficiency leads to a decrease in the transcription levels of STAT1 and STAT2, resulting in the inhibition of STAT signaling. To identify the regulators of STAT1 and STAT2, we investigated the potential role of miRNAs, which can bind to mRNAs and induce their degradation [18]. Specifically, we hypothesized that Prx II regulates the STAT signaling pathway through miRNAs. Among the candidate miRNAs, miR-221 has been shown to regulate the expression levels of STAT1 and STAT2 in glioblastoma cells [19]. Significant upregulation of miR-221 expression was observed in *Prx II* ^*−/−*^ DMSCs (Fig. 5A). To investigate whether Prx II impacts the STAT signaling pathway through a single miRNA, miR-221, or multiple miRNAs, the TarBase database was used to identify potential target miRNAs of STAT1 and STAT2. Eight target miRNAs were screened (Fig. 5B) and tested by qPCR (Fig. 5C), which revealed that there were no target miRNAs for STAT1 and STAT2. We screened 12 and 3 miRNAs using StarBase and miRDB, respectively (Fig. 5D and F), tested them using qPCR (Fig. 5E and G), and obtained results consistent with those of TarBase. Prx II likely affects the STAT signaling pathway through miR-221 alone. In WT cells, the changes in miR-221 levels were consistent with the expected changes (Supplementary Fig. 4A). Next, we treated the two cell lines with a miR-221 inhibitor and observed that STAT1 and STAT2 levels were significantly increased (Supplementary Fig. 4B). These results suggest that *Prx II* knockout inhibits the STAT signaling pathway by promoting miR-221 expression in DMSCs.

### Prx II depletion regulates Foxo1-induced miR-221 upregulation by inhibiting JNK activity

**Fig. 6:**
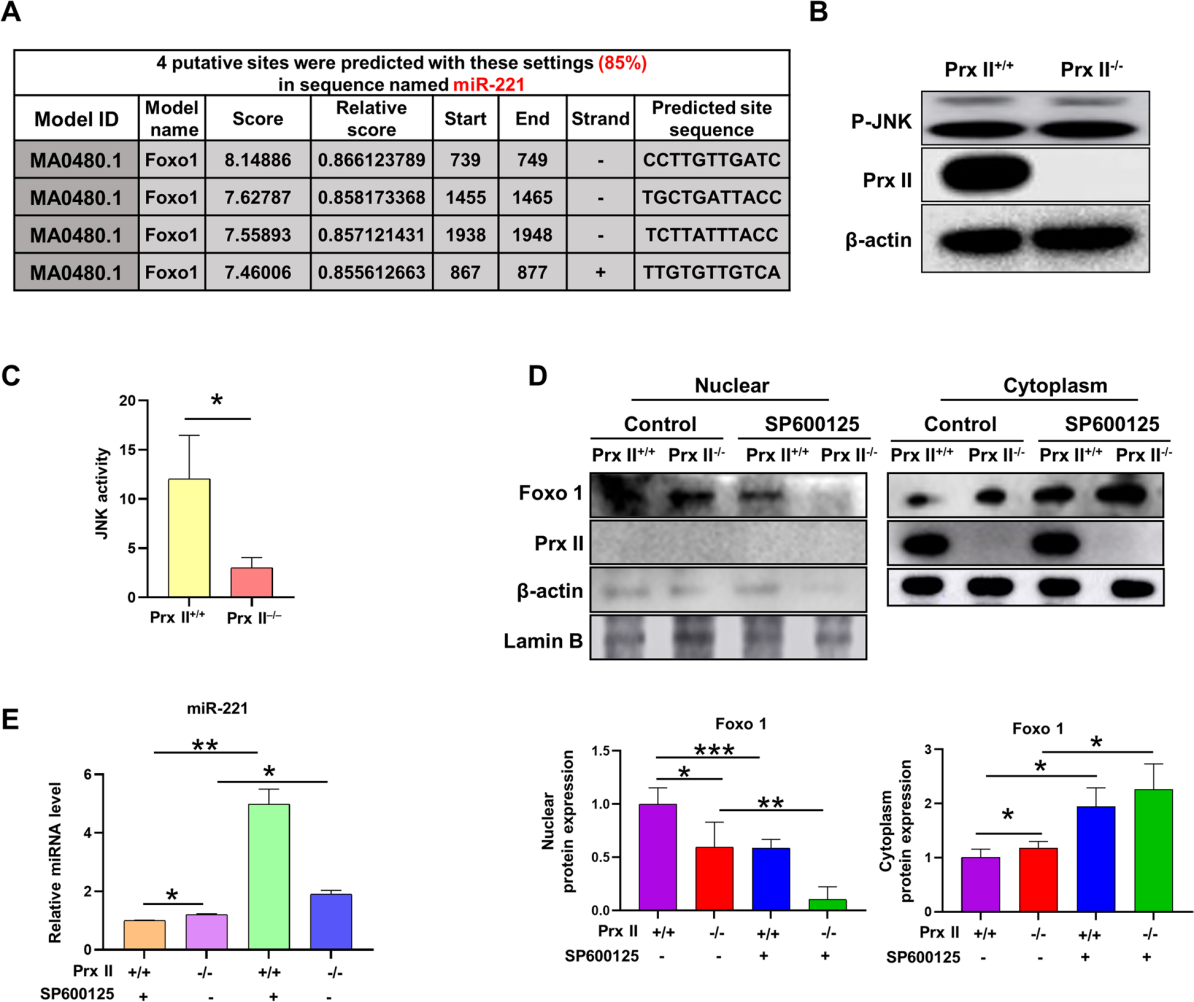
Prx II depletion regulates Foxo1-induced miR-221 upregulation by inhibiting JNK activity. **A** Binding site of Foxo1 to the miR-221 promoter. **B** Expression of phospho-JNK in *Prx II* ^*+/+*^ and *Prx II* ^*−/−*^ DMSCs. **C** Detection of JNK activation. **D** Foxo1 levels in the cell cytoplasm and nucleus were analyzed by western blotting after DMSCs were treated with 25 µM SP600125 for 48 h. Quantification of Foxo1 expression. **E** Expression of miR-221 of *Prx II* ^*+/+*^ or *Prx II* ^*−/−*^ DMSCs treated with 25µM SP600125 for 48 h. *P < 0.05, **P < 0.01, ***P < 0.001 as determined by two-tailed t test

To investigate the molecular mechanism by which Prx II regulates the expression of miR-221, we identified transcription factors (TFs). It has been reported that transcription factors can suppress the expression of miRNA by binding to the upstream promoter region of miRNA [20]. We obtained the sequence of theupstream promoter region of miR-221 from the National Center for Biotechnology Information database (https://www.ncbi.nlm.nih.gov/), and the transcription factors that could bind to this region were predicted using the JASPAR database (http://jaspar.genereg.net/). We found that these regions contained Foxo1, and its predicted binding sites are shown in Fig. 6A. We then examined the nuclear levels of Foxo1 in *Prx II* ^*+/+*^ and *Prx II* ^*−/−*^ DMSCs. Compared to *Prx II* ^*+/+*^ DMSCs, *Prx II* deletion decreased Foxo1 levels in the nucleus (Supplementary Fig. 5). JNK activation is known to affect the intracellular localization of Foxo1 [21]. However, we detected no significant difference in the levels of phosphorylated JNK was observed between *Prx II* ^*+/+*^ and *Prx II* ^*−/−*^ DMSCs (Fig. 6B). Interestingly, JNK activity was found to be significantly decreased in *Prx II* ^*−/−*^ DMSCs compared to *Prx II* ^*+/+*^ DMSCs (Fig. 6C). Additionally, treatment of DMSCs with the JNK inhibitor SP600125 resulted in a decrease in nuclear Foxo1 levels (Fig. 6D), and we observed a significant increase in miR-221 expression in *Prx II* ^*−/−*^ DMSCs treated with SP600125 (Fig. 6E). These findings suggest that Prx II depletion regulates Foxo1-induced miR-221 upregulation by inhibiting JNK activity.

#### Prx II re-expression inhibits exosome secretion by regulating ISGylation

**Fig. 7:**
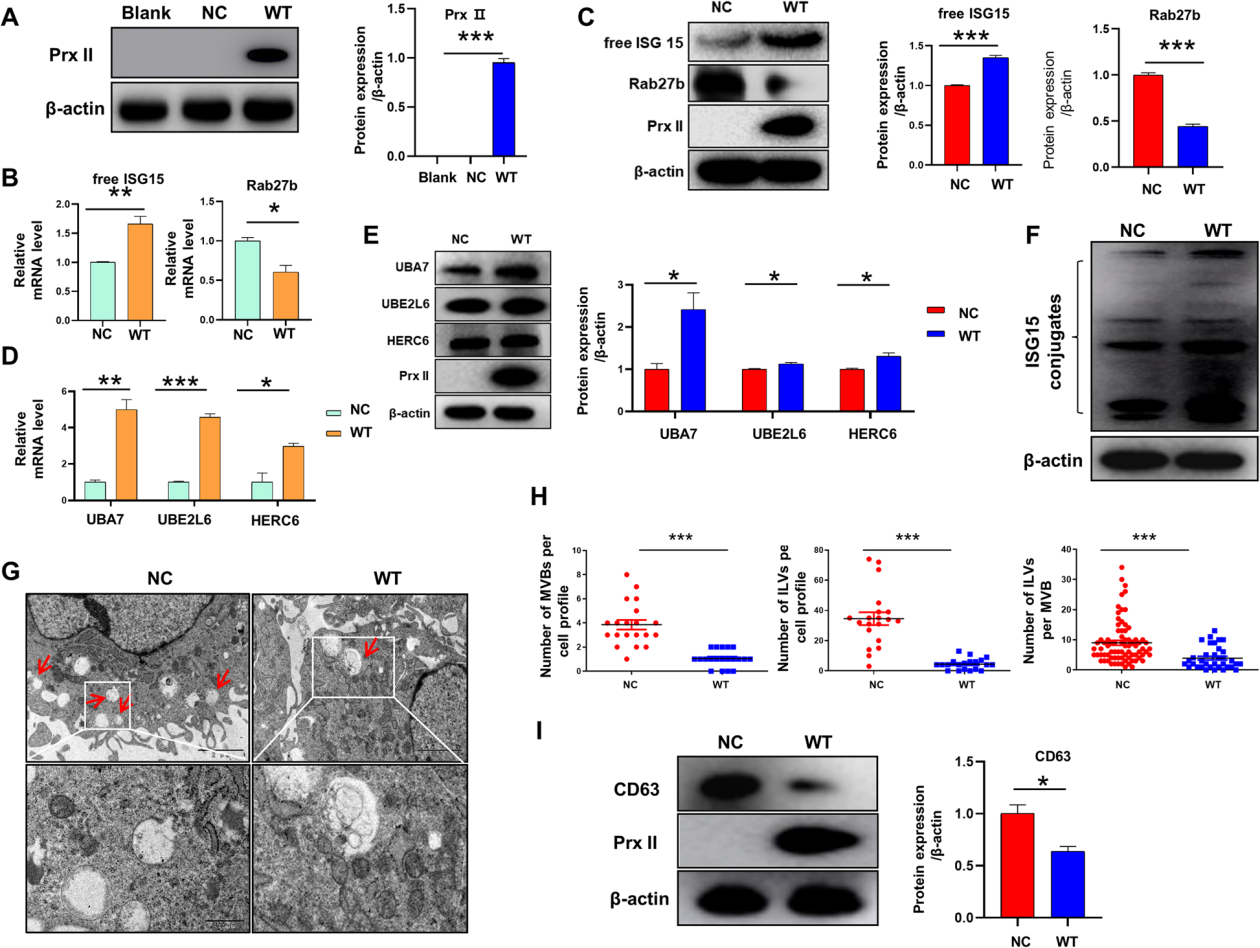
Prx II re-expression inhibits exosome secretion by regulating ISGylation. **A** Prx II expression was assessed by immunoblotting in the three groups. Quantification of Prx II expression. **B** mRNA levels of ISG15 and Rab27b in NC and WT. (In *Prx II* ^*−/−*^ DMSCs, the group transfected with blank lentiviral vectors was denoted as the NC group, whereas the group transfected with Prx II lentiviral vectors was referred to as the WT group). **C** Protein levels of ISG15 and Rab27b in NC and WT. Quantification of ISG15 and Rab27b expression. **D** Quantification of UBA7, UBE2L6, and HERC6 mRNA levels in NC and WT cells via qRT-PCR. **E** Immunoblotting and quantification of UBA7, UBE2L6, and HERC6 protein expression. **F** Conjugated ISG15 levels in NC and WT. **G** Electron micrographs showing representative fields with MVBs (red arrows) in NC or WT. **H** Numbers of MVBs, ILVs, and ILV per MVB in over 20 fields per condition. **I** CD63 expression in WT and NC was determined by western blotting and quantification. *P < 0.05, **P < 0.01, ***P < 0.001 as determined by two-tailed t test

An “add-back” rescue experiment was conducted to investigate whether Prx II regulates the expression of ISGylation-related genes, wherein GFP-labeled lentivirus carrying *Prx II* was expressed in *Prx II* knockout DMSCs (Supplementary Fig. 2). Western blotting results are presented in Fig. 7A. Our qRT-PCR analysis revealed that the mRNA levels of ISG15 and other ISGylation-related genes were upregulated in WT cells compared to Prx II knockout cells (Fig. 7B and D). Western blotting showed similar results (Fig. 7C and E), and the level of conjugated form protein of ISG15 increased (Fig. 7F). Interestingly, both mRNA and protein levels of Rab27b were decreased in WT cells, suggesting that Prx II may promote MVB degradation by reducing ISGylation, leading to a reduced expression level of RAB27B and thus regulating exosome secretion (Fig. 7B and C). To further clarify the accuracy of Prx II in regulating the numbers of MVBs, electron microscopy to quantify the number of intraluminal vesicles (ILVs) and MVBs. The results showed a decrease in the number of MVBs per cell in the WT group compared to the NC group. Meanwhile, the number of ILVs per cell profile and the number of ILVs per MVB were also decreased (Supplementary Fig. 6 and Fig. 7G and H). We also detected the expression level of the MVB marker CD63 by Western blotting, which showed that the expression level of CD63 protein in the WT group decreased compared to the NC group (Fig. 7I). Overall, these data strongly support the role of Prx II in the regulation of exosome secretion as well as the phenotype of MVBs by modulating ISGylation-related genes.

## Discussion

**Fig. 8:**
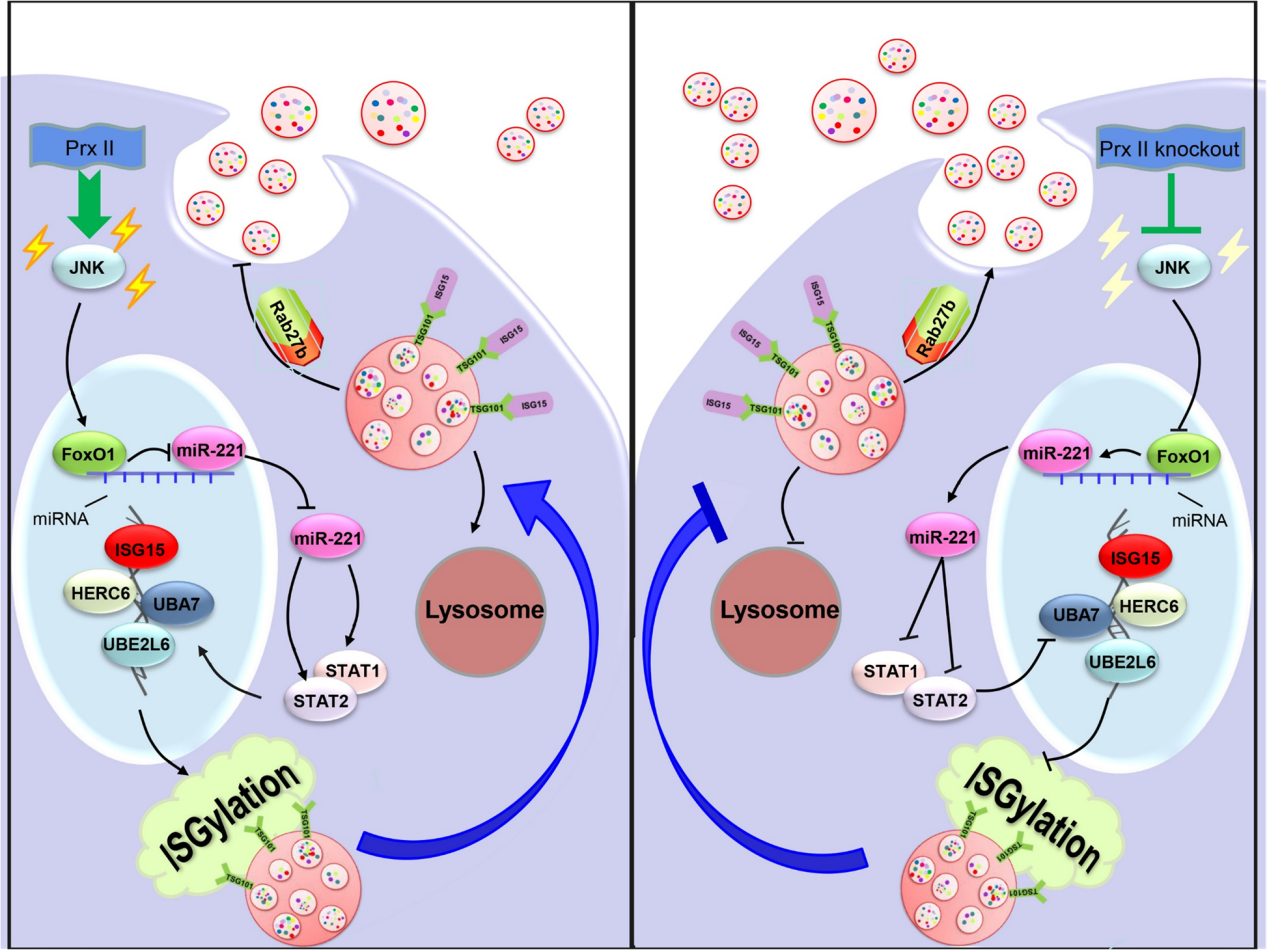
Proposed model for the role of Prx II in regulating exosome secretion from DMSCs. *Prx II* deletion results in a decrease in Foxo1 level in the nucleus by downregulating JNK activity. This decrease increases the expression of miR-221, which negatively regulates the expression of STAT1 and STAT2 to induce ISGylation, promoting exosome secretion from DMSCs

This study aimed to investigate the role of Peroxiredoxin II (Prx II) in regulating the secretion of exosomes from dermal mesenchymal stem cells (DMSCs) and elucidate the underlying molecular mechanisms. Our findings demonstrated that depletion of Prx II significantly increased the secretion of exosomes from DMSCs and increased the number of intracellular multivesicular bodies (MVBs), which are precursors of exosomes. Mechanistically, Prx II regulates the ISGylation switch, controlling MVB degradation and impairing exosome secretion. Specifically, depletion of Prx II decreased JNK activity, reduced the expression of the transcriptional inhibitor Foxo1, and promoted miR-221 expression. Increased miR-221 expression inhibited the STAT signaling pathway, downregulating the expression of ISGylation-related genes associated with MVB degradation (Fig. 8).

Currently, it is believed that exosomes are mainly secreted by their source cells through the “endocytosis-fusion-release” process. It is worth noting that after cells produce MVBs, MVBs undergo two pathways: first, MVBs bind to lysosomes under the action of ISG, degrading MVBs and their contents; second, MVBs fuse with the plasma membrane under the action of Rab27a/Rab27b releasing exosomes [4, 5]. However, the vast majority of MVBs are degraded, and only a small portion is secreted into the extracellular space as exosomes. Therefore, the upstream and downstream regulatory mechanisms of MVB degradation and intervention in MVB degradation from the source can greatly improve exosome secretion. In this study, Prx II knockout significantly increased the number of MVBs in DMSCs. It did not affect the production of MVBs but indirectly increased the expression level of Rab27b by inhibiting the ISGylation of TSG101 on MVBs, thereby inhibiting MVB degradation. This promoted the secretion of exosomes from DMSCs. A recent study has showcased that BORC (BLOC-1-related complex) in conjunction with the small GTPase ARL8 (ADP-ribosylation factor-like 8) can impede the fusion of endosomes with lysosomes. This outcome leads to the clustering of multivesicular bodies (MVBs) and subsequently amplifies the exosome secretion process. This discovery aligns with our own research, as both studies underscore the fusion between endosomes and lysosomes as a pivotal factor governing exosome secretion [22]. It is known that ISG-related genes (ISG15, UBA7, UBE2L6, and HERC6) are ISGylation; that is, their expression can be induced by the activation of the JAK/STAT signaling pathway under the stimulation of type I IFN [23]. Unlike most cells, stem cells inherently express ISGs; however, they do not produce type I IFN and have a weak response to exogenous IFN. Therefore, stem cells do not rely on typical IFN signals to express ISGs [8, 24]. miR-673 restores the IFN response of embryonic stem cells by regulating mitochondrial antiviral signaling protein [25]. In this study, we examined miRNAs that regulate the STAT signaling pathway in both cell types. miR-221 was highly expressed in Prx II knockout cells. We used a miR-221 inhibitor to demonstrate the negative regulatory relationship between miR-221, STAT1, and STAT2. Recent studies have shown that miR-221 promotes exosome secretion by downregulating the expression of PTEN, a negative regulator of exosome secretion. Our results are consistent with previous studies, and we propose a new mechanism by which miR regulates exosome secretion through the STAT signaling pathway mediated ISGylation, providing important insights into enhancing the potential therapeutic applications and efficacy of exosomes.

In this study, we also observed a decrease in the nuclear localization of Foxo1 after Prx II knockout. Through database prediction, we found that Foxo1 can promote the expression of miR-221. It has been reported that PAX3-Foxo1 can inhibit miR-221 and contribute to the pathogenesis of alveolar rhabdomyosarcoma, suggesting that Prx II regulates the expression of miR-221 through Foxo1 [21]. Activated JNK participates in the phosphorylation of 14-3-3 protein at Ser186, interfering with the cytoplasmic localization of Foxo1 [26]. We observed a decrease in JNK activity after Prx II knockout; however, JNK phosphorylation did not change. Inhibition of JNK significantly increased the expression of miR-221. Being an antioxidant enzyme, Prx II is postulated to govern JNK activity by influencing ROS (reactive oxygen species) levels. Research has indicated that elevated ROS levels can trigger the activation of ATM (ataxia-telangiectasia mutated) and Erk (extracellular signal-regulated kinase) pathways. These pathways, in turn, can hinder lysosomal acidification, leading to inadequate degradation of multivesicular bodies (MVBs) by inhibiting vacuolar ATPase (vATPase) [27]. We also assessed the ROS levels in both cell types; however, no significant differences were observed. Therefore, additional investigations are warranted to comprehensively elucidate the role of Prx II in the regulation of JNK activity.

Most miRNAs mainly exert regulatory functions by inhibiting translation and inducing mRNA degradation; however, some reports have shown that miRNAs can positively regulate gene expression under specific conditions. miR-10a interacts with the 5’ untranslated region of mRNA encoding ribosomal proteins, enhancing translation [28]. The downregulation of miR-148b-3p, miR-214-3p, miR-19b-3p, miR-15a-5p, miR-15b-5p, miR-16-5p, and miR-155-5p after Prx II knockout is an interesting finding. Whether they regulate the STAT signaling pathway and participate in regulating exosome secretion will be of interest for future research. Additionally, Prx II is a peroxidase that participates in various biological processes in cells, and whether Prx II knockdown affects other biological processes and thus the secretion of exosomes by DMSCs needs to be further investigated.

Exosomes play a pivotal role in intercellular communication, and comprehending the regulatory mechanisms involving Prx II in exosome secretion holds the promise of refining the composition and functionality of exosomes. This optimization can enhance their effectiveness in tissue repair and regeneration, thereby offering novel therapeutic strategies within the realm of clinical regenerative medicine.

While exosomes exhibit significant potential for clinical applications, there remain certain risks and challenges associated with their use. These challenges encompass aspects such as the safety of exosome therapy, concerns related to potential tumorigenesis, and ethical considerations. To ensure the secure and effective implementation of exosome therapy, further dedicated research and meticulous clinical trials are indispensable.

## Conclusion

In Conclusion, this study provides important insights into the molecular mechanisms regulating exosome secretion in DMSCs and highlights the critical role of Prx II in controlling the ISGylation switch that regulates DMSC-exosome secretion. These findings have significant implications for developing new therapeutic strategies for lung injury and other diseases. However, further research is needed to fully elucidate the complex regulatory mechanisms of exosome secretion and explore the potential therapeutic applications of DMSC-derived exosomes.

### Supplementary Information

**Additional file 1: Supplementary Fig 1.** (A) Electron micrographs showing MVBs in *Prx II*^+/+^and *Prx II*^−/−^DMSCs. Red arrows indicate MVBs containing typical intraluminal vesicles (ILVs).** Supplementary Fig 2.** Re-expression of Prx II in *Prx II*^−/−^DMSCs. (A) GFP expression in blank (*Prx II*^−/−^DMSCs), NC (*Prx II*^−/−^DMSCs transfected with empty lentiviral particles), and WT (*Prx II*^−/−^DMSCs transfected with lentiviral particles containing *Prx II* re-expression sequence) was observed under a fluorescence microscope. (B) Flow cytometry analysis of the cells in the three groups. **Supplementary Fig 3.** Re-expression of Prx II in *Prx II*^−/−^DMSCs promotes STAT signaling. (A) Protein levels of phospho-STAT1, STAT1, phospho-STAT2, and STAT2 in NC and WT. (B) mRNA levels of STAT1 and STAT2 in NC and WT. **Supplementary Fig 4.** Re-expression of Prx II promotes the STAT signaling pathway by inhibiting miR-221. (A) qRT-PCR analysis of miR-221 expression in NC and WT. (B) Western blot analysis of STAT1 and STAT2 levels in transfected control (NC) or cells transfected with the miR-221 inhibitor for 48 h. **Supplementary Fig 5. ***PrxII* knockdown inhibits FOXO1 entry into the nucleus.(A) Foxo1 levels in the nucleus were determined via western blotting. **Supplementary Fig 6.** (A) Electron micrographs showing representative fields with MVBs (red arrows) in NC or WT

## Data Availability

All data that support the findings of this study are available from the corresponding authors upon reasonable request.

## Abbreviations

ISGs: Interferon-stimulated genes
Prx II: Peroxiredoxin II
DMSCs: Dermal mesenchymal stem cells
MVBs: Multivesicular bodies
miRNAs: microRNAs
mRNAs: messenger RNAs
ILVs: Intraluminal vesicles
Prxs: Peroxiredoxins
DMSC-Exos: Dermal mesenchymal stem cell-exosomes
JNK: c-Jun N-terminal kinase
Foxo1: Forkhead box O 1
STAT: Signal Transducers and Activators of Transcription
