# Supplementary material for: Peroxiredoxin II regulates exosome secretion from dermal mesenchymal stem cells through the ISGylation signaling pathway
Source: Cell Commun Signal. 2023 Oct 20;21:296. doi: 10.1186/s12964-023-01331-w (PMC10588245; doi:10.1186/s12964-023-01331-w)
Supplement: Supplementary file 2 — Additional file 1: Supplementary Fig 1. (A) Electron micrographs showing MVBs in Prx II+/+and Prx II−/−DMSCs. Red arrows indicate MVBs containing typical intraluminal vesicles (ILVs). Supplementary Fig 2. Re-expression of Prx II in Prx II−/−DMSCs. (A) GFP expression in blank (Prx II−/−DMSCs), NC (Prx II−/−DMSCs transfected with empty lentiviral particles), and WT (Prx II−/−DMSCs transfected with lentiviral particles containing Prx II re-expression sequence) was observed under a fluorescence microscope. (B) Flow cytometry analysis of the cells in the three groups. Supplementary Fig 3. Re-expression of Prx II in Prx II−/−DMSCs promotes STAT signaling. (A) Protein levels of phospho-STAT1, STAT1, phospho-STAT2, and STAT2 in NC and WT. (B) mRNA levels of STAT1 and STAT2 in NC and WT. Supplementary Fig 4. Re-expression of Prx II promotes the STAT signaling pathway by inhibiting miR-221. (A) qRT-PCR analysis of miR-221 expression in NC and WT. (B) Western blot analysis of STAT1 and STAT2 levels in transfected control (NC) or cells transfected with the miR-221 inhibitor for 48 h. Supplementary Fig 5. PrxII knockdown inhibits FOXO1 entry into the nucleus.(A) Foxo1 levels in the nucleus were determined via western blotting. Supplementary Fig 6. (A) Electron micrographs showing representative fields with MVBs (red arrows) in NC or WT [file 12964_2023_1331_MOESM1_ESM.docx]

**Supplementary Figures.**

**
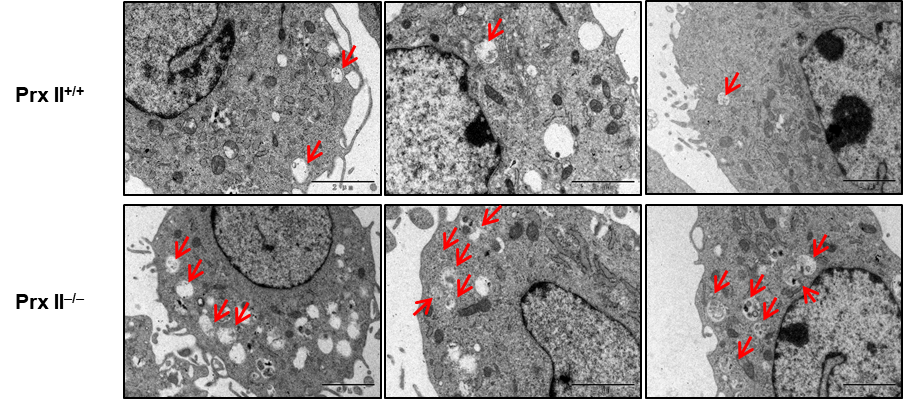
**

**Supplementary Fig. 1.** (A) Electron micrographs showing MVBs in *Prx II^+/+^* and *Prx II^−/−^* DMSCs. Red arrows indicate MVBs containing typical intraluminal vesicles (ILVs).


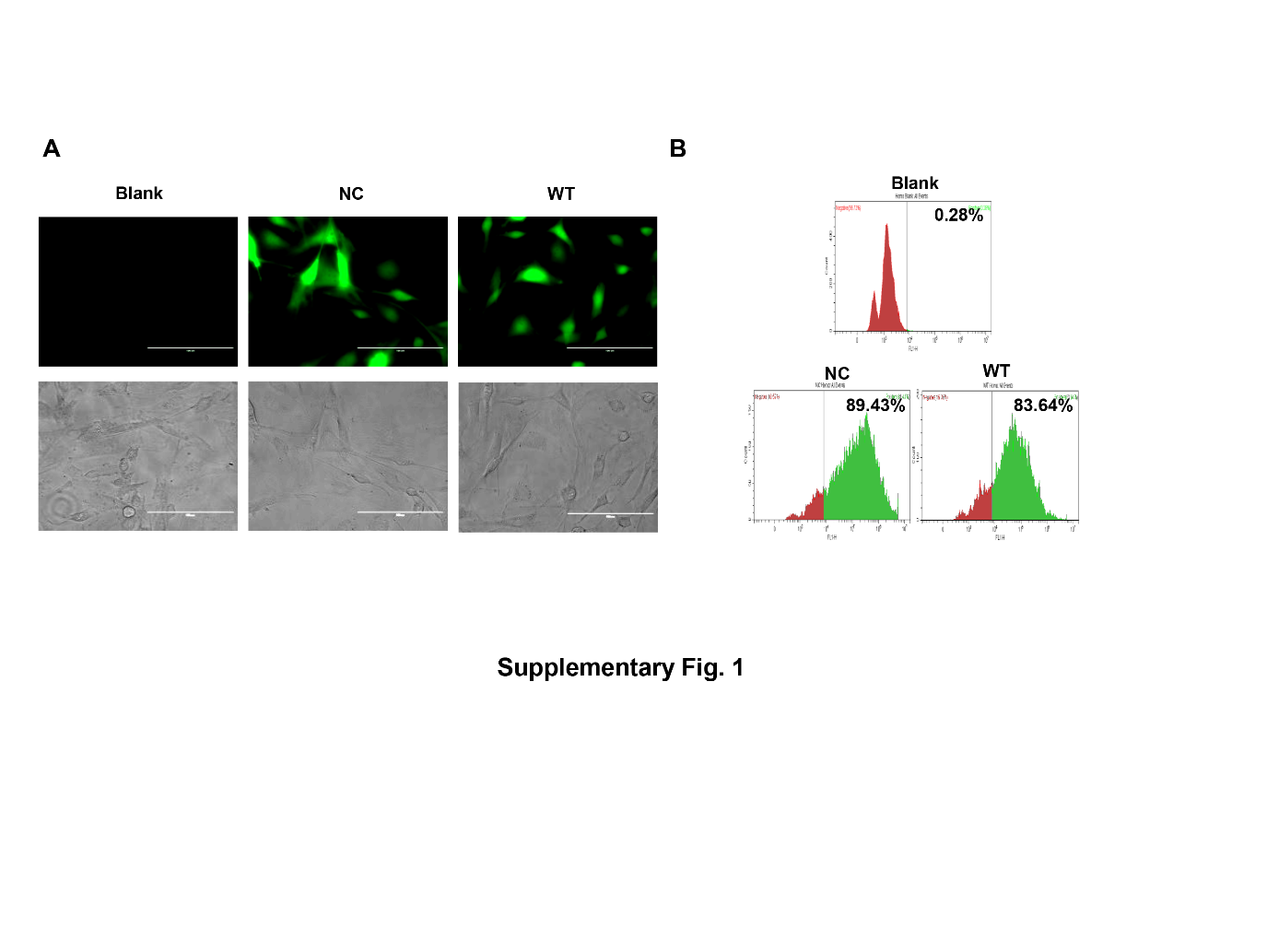


**Supplementary Fig. 2.** Re-expression of Prx II in *Prx II^−/−^* DMSCs. (A) GFP expression in blank (*Prx II^−/−^* DMSCs), NC (*Prx II^−/−^* DMSCs transfected with empty lentiviral particles), and WT (*Prx II^−/−^* DMSCs transfected with lentiviral particles containing *Prx II* re-expression sequence) was observed under a fluorescence microscope. (B) Flow cytometry analysis of the cells in the three groups.


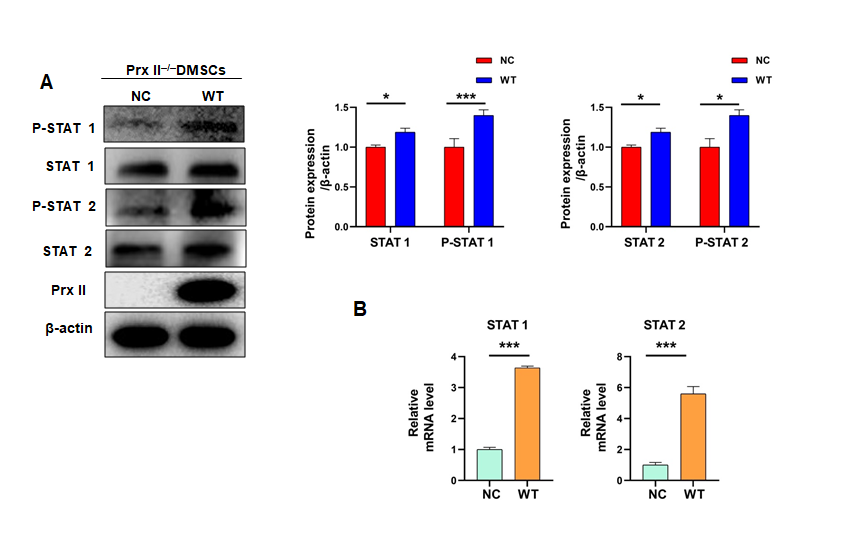


**Supplementary Fig. 3.** Re-expression of Prx II in *Prx II^−/−^* DMSCs promotes STAT signaling. (A) Protein levels of phospho-STAT1, STAT1, phospho-STAT2, and STAT2 in NC and WT. (B) mRNA levels of STAT1 and STAT2 in NC and WT.


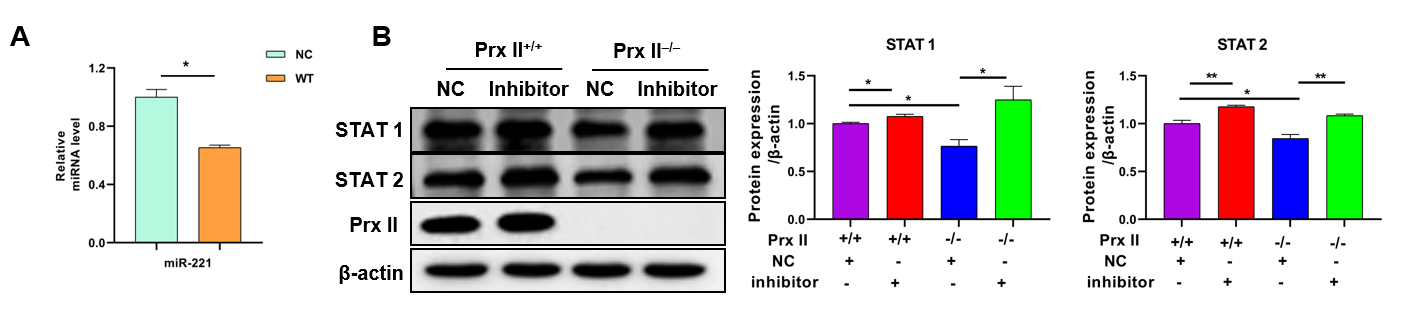


**Supplementary Fig. 4.** Re-expression of Prx II promotes the STAT signaling pathway by inhibiting miR-221. (A) qRT-PCR analysis of miR-221 expression in NC and WT. (B) Western blot analysis of STAT1 and STAT2 levels in transfected control (NC) or cells transfected with the miR-221 inhibitor for 48 h.


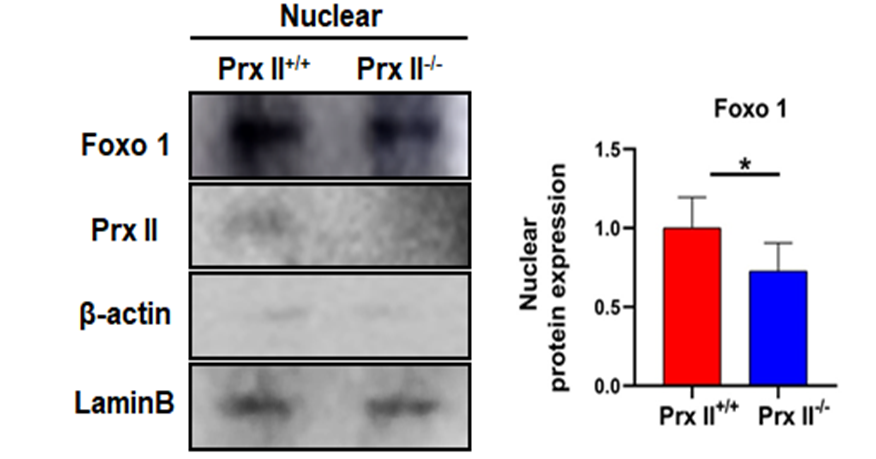


**Supplementary Fig. 5.** *PrxII* knockdown inhibits FOXO1 entry into the nucleus. (A) Foxo1 levels in the nucleus were determined via western blotting.


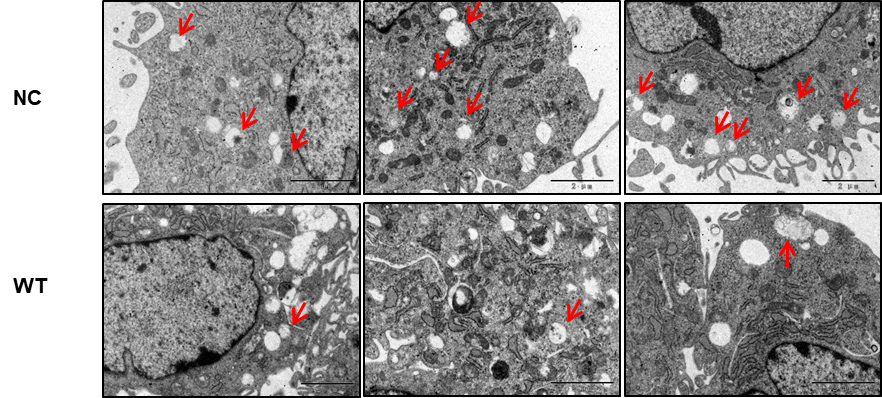


**Supplementary Fig. 6.** (A) Electron micrographs showing representative fields with MVBs (red arrows) in NC or WT.
